# A pilot crossover study: effects of an intervention using an activity monitor with computerized game functions on physical activity and body composition

**DOI:** 10.1186/1880-6805-33-35

**Published:** 2014-12-02

**Authors:** Masato Nishiwaki, Akinori Kuriyama, Yumi Ikegami, Nana Nakashima, Naoyuki Matsumoto

**Affiliations:** Faculty of Engineering, Osaka Institute of Technology, 5-16-1 Ohmiya, Asahi-Ku, Osaka 535-8585 Japan; Faculty of Environmental Symbiotic Sciences, Prefectural University of Kumamoto, 3-1-100 Tsukide, Higashi-ku, Kumamoto 862-8502 Japan

**Keywords:** Amount of physical activity, Steps, Yuuhokei, Lifecorder EX, Body fat, Health, Physical Activity Reference for Health Promotion 2013

## Abstract

**Background:**

Wearing an activity monitor as a motivational tool and incorporating a behavior-based reward system or a computerized game element might have a synergistic effect on an increase in daily physical activity, thereby inducing body fat reduction. This pilot crossover study aimed to examine the effects of a short-term lifestyle intervention using an activity monitor with computerized game functions on physical activity and body composition.

**Methods:**

Twenty healthy volunteers (31 ± 3 years) participated in a 12-week crossover study. The participants were randomly assigned to either Group A (a 6-week game intervention followed by a 6-week normal intervention) or Group B (a 6-week normal intervention followed by a 6-week game intervention). The participants wore both a normal activity monitor (Lifecorder EX) and an activity monitor with computerized game functions (Yuuhokei) during the game intervention, whereas they only wore a normal activity monitor during the normal intervention. Before, during, and after the intervention, body composition was assessed.

**Results:**

Significantly more daily steps were recorded for the game intervention than for the normal intervention (10,520 ± 562 versus 8,711 ± 523 steps/day, *P* < 0.01). The participants performed significantly more physical activity at an intensity of ≥ 3 metabolic equivalents (METs) in the game intervention than in the normal intervention (3.1 ± 0.2 versus 2.4 ± 0.2 METs · hour/day, *P* < 0.01). Although body mass and fat were significantly reduced in both periods (*P* < 0.01), the difference in body fat reduction was significantly greater in the game intervention than in the normal intervention (*P* < 0.05).

**Conclusions:**

A short-term intervention using an activity monitor with computerized game functions increases physical activity and reduces body fat more effectively than an intervention using a standard activity monitor.

## Background

Higher levels of physical activity and exercise are associated with lower risk of non-communicable diseases, such as cardiovascular diseases [1, 2]. Adults in developed countries have been recommended to perform 30 minutes of moderate to vigorous daily physical activity [3]. The Japan Ministry of Health, Labour, and Welfare has also published *Physical Activity Reference for Health Promotion 2013*, which recommends increasing physical activity by an additional 10 minutes every day, anywhere, anytime [4]. However, technological developments such as the Internet and cellular phones have tended to reduce daily physical activity as less energy is needed for routine daily activities [1, 5, 6]. Therefore, the importance of increased daily physical activity has been emphasized to prevent or to reduce the incidence of lifestyle-related diseases.

A meta-analysis of 8 randomized controlled trials and 18 observational studies found that physical activity significantly increased among pedometer users by about 2,000 to 2,500 steps/day over control participants or baseline [7]. These findings suggested that the immediate feedback provided by the activity monitor is an important motivational feature that serves as a behavioral modification tool [8]. Therefore, in addition to being a surveillance tool, activity monitors are also popular motivational devices. Indeed, many studies have indicated that lifestyle intervention using activity monitors can increase regular physical activity and help the prevention of lifestyle-related diseases [7, 9–11].

Previous studies have reported on lifestyle interventions using behavior-based reward systems [12, 13]. Interestingly, open-loop feedback intervention, such as using an activity monitor and TV viewing time as a reward, induces an increase in physical activity in children [13]. Systematic review also has indicated that active video games increase energy expenditure compared to rest or playing passive video games [14]. Because participants become absorbed in playing interactive video games and perceive them as enjoyable, active video games can serve as an additional intervention to encourage compliance with exercise or rehabilitation programs [15–18]. Therefore, lifestyle intervention using a behavior-based reward system or a computerized game element may promote daily physical activity or reduce sedentary behaviors.

Recent evidence indicates that an increase in physical activity without caloric restriction is a useful strategy for reducing obesity; in particular, abdominal and visceral obesity. Furthermore, an increase in physical activity is generally dose-dependently associated with a reduction in body fat within a relatively short intervention of 16 weeks [19]. Thus, if lifestyle intervention using a behavior-based reward system or a computerized game element promotes daily physical activity, significant reductions in body weight and fat would also be observed.

Based on this information, wearing an activity monitor as a motivational tool and incorporating a behavior-based reward system or a video game element might have a synergistic effect on daily physical activity, such as the number of steps and amount of physical activity (amount of PA). Thus, we hypothesized that a short-term lifestyle intervention using an activity monitor with computerized game functions would increase daily physical activity more than normal intervention using an activity monitor. In addition, we hypothesized that more fat would be lost via an intervention using an activity monitor with computerized game functions than a standard intervention. Therefore, the present pilot study primarily aimed to examine the effects of a short-term lifestyle intervention using an activity monitor with computerized game functions on daily physical activity and body composition.

## Methods

### Participants

Twenty Japanese adults in Kumamoto, Japan (age, 20 to 49 years) without chronic diseases that could affect cardiovascular, metabolism or daily physical activity participated in this study. Participants were recruited from among the students and staff at the Prefectural University of Kumamoto. None of the participants had performed any endurance or resistance training on a regular basis. They were matched for physical characteristics, such as age and body mass index (BMI), and then randomized to either Group A (n = 10) or Group B (n = 10) (Table 1). All participants provided written informed consent to participate after receiving an explanation about the study procedures and risks. The Human Ethics Committee at the Prefectural University of Kumamoto approved the protocol.

**Table 1:** **Physical characteristics of participants**

Variables		All	Group A	Group B
Number of subjects	(men/women)	20 (6/14)	10 (3/7)	10 (3/7)
Age	(years)	31 ± 3	32 ± 3	31 ± 4
Height	(cm)	160.9 ± 2.2	160.7 ± 3.9	161.1 ± 2.5
Body mass	(kg)	56.5 ± 2.7	56.9 ± 3.9	56.2 ± 3.8

Group A, game intervention for six weeks followed by normal intervention for six weeks; Group B, normal intervention for six weeks followed by game intervention for six weeks.

### Experimental procedures

Body composition and blood pressure (BP) were measured on all participants before (baseline), during (after 6 weeks; the intervention’s midpoint) and at the end (after 12 weeks) of the lifestyle intervention. All tests were conducted in a quiet, air-conditioned room (22 to 24 °C) at the same time of day and at the same number of hours after the last meal throughout the study period to avoid potential diurnal variations. The participants abstained from caffeine and fasted for > 4 hours before each test.

### Body composition and BP

Measurements were conducted based on standard procedures [20, 21]. Body composition was determined using the bioelectric impedance method (TBF-410, Tanita Co., Tokyo, Japan). It has been reported that the instrumental method can accurately detect body composition changes and that the validity of body composition assessment is high [22–25]. BMI was calculated as body mass divided by square of height. BP and pulse rates were also determined using an automated sphygmomanometer (HEM-7080IC, Omron Co., Kyoto, Japan). The day-to-day coefficients of variations (CV) for body mass, body fat, and BP were all < 10%.

### Lifestyle intervention

A short-term pilot lifestyle intervention was performed. The 12 weeks crossover study comprised the game intervention (G) period and the normal intervention (N) period. The participants wore both a normal activity monitor and an activity monitor with computerized game functions during the G period, whereas they only wore a normal activity monitor during the N period. Group A participated in 6 weeks of G followed by 6 weeks of N; while Group B participated in 6 weeks of N followed by 6 weeks of G. The normal activity monitor was the Lifecorder EX (Suzuken Co., Aichi, Japan) and the activity monitor with computerized game functions was the Yuuhokei (Bandai Co., Tokyo, Japan). Throughout the lifestyle intervention, participants were encouraged to walk above 10,000 steps/day counted by the Lifecorder EX [9, 10]. To confirm physical activity levels, data were recorded every week throughout both of the experimental periods and provided to the participants as feedback. During both periods, Lifecorder EX data downloaded for personal computer (PC) in each participant’s workplace once a week. Based on weekly physical activity data, the participants were advised by a specialized exercise instructor on how to increase daily physical activity (that is take a brisk walk while commuting, use the stairs, and go shopping by walking).

In addition to the above lifestyle intervention, the Yuuhokei was utilized for further increases in physical activity in the G period. The Yuuhokei has a role-playing game, and is fundamentally a computerized game more than an activity monitor. The games included were Space Battleship Yamato (n = 7), The Rose of Versailles (n = 6), Section Chief Kousaku Shima (n = 4), and Unification of the Whole Country (n = 3). These are based on stories and tales that are very famous in Japan. All study participants knew almost all of the game stories or tales at the start of the intervention, because they had seen them as televised cartoons or read them as comics during childhood. The participants selected their preferred game story and were encouraged to clear the preferred game. Although the number of steps measured by the Yuuhokei was significantly lower than that measured by the Lifecorder EX [26], a strongly significant correlation was observed between the step counts obtained from the Yuuhokei and the Lifecorder EX (r = 0.92). Therefore, in Yuuhokei, a target value for each game to proceed to the next scene was set at 8,000 steps for all participants, which approximately corresponds to 10,000 steps counted by the Lifecorder EX [26]. During the G period, after reaching the required amount of steps in Yuuhokei, the story or stage of the role-playing game progresses. The participants were able to attempt to increase the amount of steps required to clear the stage of the game and were also to read a success story relating the game in detail, at any time of the day. When the participants constantly perform or accumulate 8,000 steps counted by the Yuuhokei, game characters considerably praise the participants. Conversely, when they constantly cannot perform or accumulate 8,000 steps, game characters encourage them to increase physical activity and to clear the current scene of the game. In order to clear each stage, participants were required to walk.

### Assessment of the physical activity amount

The physical activity data (steps and amount of PA) from the Lifecorder EX (a single-axis activity monitor) were used for analysis [27–32]. The Yuuhokei was used only as a gaming device to motivate participants. The accuracy of the step counts detected by Lifecorder EX is calibrated during the manufacturing process according to the Japanese Industrial Standards (JIS), and the widely accepted error during treadmill walking is within 1% [27–29]. Activity monitors were set up by observers based on the height and body mass of each participant, who then attached the devices to the side of the waist at the midline of the left thigh [27–29] and the participants wore them constantly except for dressing, bathing, and sleeping. The participants also reported when they did not wear the devices [28, 29].

After the lifestyle intervention, step counts (steps/day) and the amount of time spent at respective activity intensities detected by the Lifecorder EX (the devise records a signal of 0, 0.5, or 1 to 9 every 4 seconds while being worn; signals/day) were downloaded using Physical Activity Analysis Software (Version 2, Suzuken Co., Aichi, Japan). The relationship between metabolic equivalents (METs) and the activity intensity is considered highly significant (r^2^ = 0.929), and thus activity intensities were converted into METs according to the methods of Kumahara *et al*. [27]. Because the guidelines of the American College of Sports Medicine (ACSM) or the American Heart Association (AHA) focus on 30 minutes of moderate-intensity (3.0 to- 6.0 METs) daily physical activity 5 days/week or vigorous-intensity (> 6 METs) aerobic activity for a minimum of 20 minutes for 3 days/week [3], the daily amount of PA at the intensity of 3 METs or more (that is METs · hour/day) was determined, and then the mean values were calculated for each N and each G. The average step counts and amount of PA per week were also calculated to determine the time course of changes in steps and amount of PA. As previously reported [33], we only used the data from when the activity monitor was worn continuously while waking throughout the day until going to bed, and days that the equipment was not worn were excluded from the analysis [29].

### Statistical analysis

Data are presented as means ± standard error of the mean (SEM). Parameters before lifestyle intervention were compared between the two groups using an independent Student’s *t*-test. Changes in daily physical activity were analyzed using 2-way (group (Group A versus Group B) × intervention (N versus G)) and 3-way (group (Group A versus Group B) × intervention (N versus G) × time (1 to 6 weeks)) repeated-measures analysis of variance (ANOVA). The effects of intervention on physiological variables of body composition and BP were analyzed by three-way (group (Group A versus Group B) × intervention (G versus N) × time (Pre versus Post)) repeated-measures ANOVA. The Tukey method was used for *post hoc* multiple comparisons when F values were significant. Changes between Pre- and Post-intervention body composition were assessed using a dependent Student’s *t*-test. Results were regarded as statistically significant if *P* < 0.05.

## Results

Baseline physical characteristics did not significantly differ between the groups before the lifestyle intervention (Table 1).

**Figure 1:**
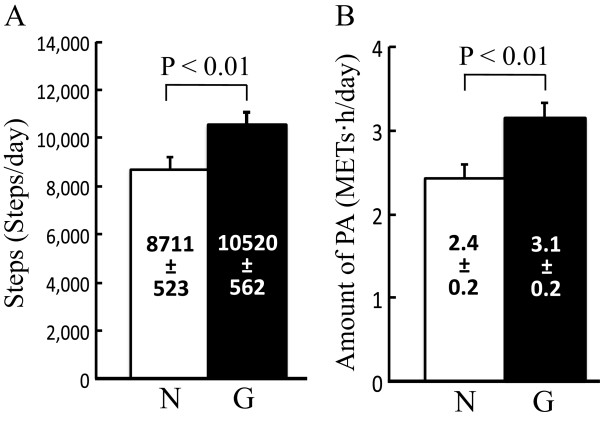
**Comparisons of physical activity between normal (N) and game (G) interventions.** Comparisons of daily steps **(A)** and amount of daily physical activity at intensity of ≥ 3 (metabolic equivalents) METs **(B)**. N, period of normal intervention; G, period of intervention using an activity monitor with game functions; PA, physical activity. Data are shown as means ± standard error of the mean (SEM).

The number of daily steps was significantly higher in G than in N throughout the lifestyle intervention (*P* < 0.01; Figure 1A). The amount of PA (daily total METs · hour) at an intensity of ≥ 3 METs was significantly higher in G than in N (*P* < 0.01; Figure 1B). We found no significant main effects of group (steps, *P* = 0.157; amount of PA, *P* = 0.863) or interactions (steps, *P* = 0.799; amount of PA, *P* = 0.388). The mean differences in steps and amount of PA between G and N interventions were 1,809 ± 407 steps (23.9 ± 5.6%) and 0.7 ± 0.2 METs · hour (36.5 ± 8.9%), respectively. In addition, no statically significant differences were found in steps and amount of PA among the four game stories installed in Yuuhokei.

We further analyzed the time course of changes in steps and amount of PA. Although the numbers of steps and amount of PA were significantly higher in G than in N (*P* < 0.05), the time course did not significantly change for either the number of steps (*P* = 0.714) or amount of PA (*P* = 0.196) (Table 2).

**Table 2:** **Comparisons of time course of change in physical activity between normal and game interventions**

	1	2	3	4	5	6
Steps/day						
Normal	9,005 ± 501	8,864 ± 614	8,839 ± 516	8,452 ± 511	9,046 ± 573	8,218 ± 627
Game	10,039 ± 699	10,471 ± 540	10,387 ± 550	11,039 ± 597	10,562 ± 530	10,339 ± 616
Three-way ANOVA						
Group	Intervention	Time	Interaction (G × I)	Interaction (G × T)	Interaction (I × T)	Interaction (G × I × T)
*P* = 0.063	*P* = 0.028	*P* = 0.714	*P* = 0.889	*P* = 0.9012	*P* = 0.308	*P* = 0.0386
PA (METs hour)/day						
Normal	2.6 ± 0.3	2.5 ± 0.2	2.5 ± 0.2	2.3 ± 0.2	2.6 ± 0.3	2.1 ± 0.2
Game	3.0 ± 0.3	3.1 ± 0.2	2.8 ± 0.2	3.3 ± 0.3	3.4 ± 0.2	3.0 ± 0.2
Three-way ANOVA						
Group	Intervention	Time	Interaction (G × I)	Interaction (G × T)	Interaction (I × T)	Interaction (G × I × T)
*P* = 0.832	*P* = 0.033	*P* = 0.196	*P* = 0.645	*P* = 0.593	*P* = 0.252	*P* = 0.196

ANOVA, analysis of variance; *G*, group; I, intervention; METs, metabolic equivalents; T, time.

**Figure 2:**
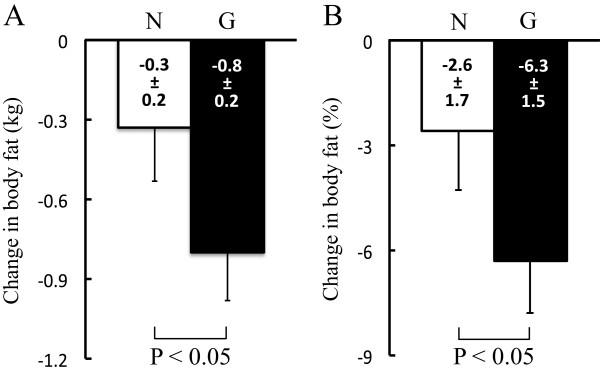
**Comparisons of the reduction of body fat.** Comparisons of absolute **(A)** and relative **(B)** changes in body fat. Figure format is the same as Figure 1.

To determine the effects of each intervention method on body composition and BP, physiological parameters were analyzed. Group (Group A versus Group B) × intervention (G versus N) × time (Pre versus Post) ANOVA revealed a significant reduction in body mass (*P* < 0.01), body fat (*P* < 0.01), and BMI (*P* < 0.01) over time for both G and N interventions (Table 3). However, significantly more fat was lost in G than in N (*P* < 0.05) (Figures 2A, B). Lean body mass did not significantly change in either intervention (Table 3). Systolic BP was only slightly reduced after both interventions, but the difference reached statistical significance (*P* < 0.05; Table 3).

**Table 3:** **Body composition and blood pressure before and after intervention**

Variables		Pre	Post		Three-way ANOVA		
Body mass (kg)	N	56.0 ± 2.6	55.5 ± 2.7	Group	Intervention	Time	Interaction (G × I × T)
	G	56.1 ± 2.6	55.4 ± 2.5	*P* = 0.879	*P* = 0.998	*P* = 0.001	*P* = 0.064
					Interaction (G × I)	Interaction (G × T)	Interaction (I × T)
					*P* = 0.879	*P* = 0.964	*P* = 0.5030
Lean body mass (kg)	N	43.1 ± 2.0	42.9 ± 2.1	Group	Intervention	Time	Interaction (G × I × T)
	G	43.0 ± 2.1	43.1 ± 2.1	*P* = 0.637	*P* = 0.976	*P* = 0.914	*P* = 0.0201
					Interaction (G × I)	Interaction (G × T)	Interaction (I × T)
					*P* = 0.9958	*P* = 0.5524	*P* = 0.331
Body fat percent (%)	N	23.2 ± 0.8	22.8 ± 0.9	Group	Intervention	Time	Interaction (G × I × T)
	G	23.4 ± 0.9	22.3 ± 1.0	*P* = 0.314	*P* = 0.914	*P* = 0.002	*P* = 0.444
					Interaction (G × I)	Interaction (G × T)	Interaction (I × T)
					*P* = 0.561	*P* = 0.542	*P* = 0.108
Body fat mass (kg)	N	13.0 ± 0.8	12.7 ± 0.8	Group	Intervention	Time	Interaction (G × I × T)
	G	13.1 ± 0.8	12.3 ± 0.8	*P* = 0.493	*P* = 0.938	*P* = 0.002	*P* = 0.719
					Interaction (G × I)	Interaction (G × T)	Interaction (I × T)
					*P* = 0.6262	*P* = 0.519	*P* = 0.097
Body mass index	N	21.4 ± 0.5	21.2 ± 0.5	Group	Intervention	Time	Interaction (G × I × T)
	G	21.5 ± 0.5	21.2 ± 0.5	*P* = 0.769	*P* = 0.971	*P* = 0.0005	*P* = 0.076
					Interaction (G × I)	Interaction (G × T)	Interaction (I × T)
					*P* = 0.754	*P* = 0.658	*P* = 0.495
Systolic BP (mmHg)	N	112 ± 2	110 ± 2	Group	Intervention	Time	Interaction (G × I × T)
	G	113 ± 2	111 ± 2	*P* = 0.887	*P* = 0.721	*P* = 0.045	*P* = 0.003
					Interaction (G × I)	Interaction (G × T)	Interaction (I × T)
					*P* = 0.491	*P* = 0.291	*P* = 0.638
Diastolic BP (mmHg)	N	70 ± 2	70 ± 2	Group	Intervention	Time	Interaction (G × I × T)
	G	70 ± 2	69 ± 2	*P* =0.631	*P* = 0.782	*P* = 0.677	*P* = 0.060
					Interaction (G × I)	Interaction (G × T)	Interaction (I × T)
					*P* = 0.878	*P* = 0.452	*P* = 0.694
Pulse pressure (mmHg)	N	42 ± 2	40 ± 2	Group	Intervention	Time	Interaction (G × I × T)
	G	44 ± 2	42 ± 2	*P* = 0.690	*P* = 0.463	*P* = 0.081	*P* = 0.257
					Interaction (G × I)	Interaction (G × T)	Interaction (I × T)
					*P* = 0.535	*P* = 0.040	*P* = 0.967
Pulse rate (beats/minute)	N	67 ± 2	66 ± 2	Group	Intervention	Time	Interaction (G × I × T)
	G	67 ± 2	67 ± 2	*P* = 0.223	*P* = 0.910	*P* = 0.723	*P* = 0.844
					Interaction (G × I)	Interaction (G × T)	Interaction (I × T)
					*P* = 0.865	*P* = 0.813	*P* = 0.813

BP, blood pressure; Pre, before intervention; Post, after intervention. Figure Abbreviations are the same as Figure 2 and Table 2.

## Discussion

This is the pilot study to clarify the effects of a lifestyle intervention using an activity monitor with computerized game functions on daily physical activity and body composition. The most important findings were that the number of steps and amount of PA were significantly higher and significantly more body fat was lost in the game than in the normal intervention.

Previous studies of pedometer reactivity (that is activity changes as a results of wearing a pedometer) have found a significant increase in mean daily step counts (approximately 1,800 to 1,000 steps/day) when participants wear an unsealed pedometer and record their daily step counts in an activity log, compared with wearing a covert (uninformed device and measured values) or a sealed (uninformed measured values) pedometer [8, 34]. Accordingly, these results indicate that being conscious of wearing an activity monitor and confirming the measured values induce an increase in daily step counts. Although our participants did not log daily steps in an activity diary, observers recorded their data every week throughout the experimental period and feedback was constantly provided to the participants. Such feedbacks are very likely to induce a slight increase in daily physical activity as a motivational effect [8]. Step counts and amount of PA during N period may thus be slightly higher than usual in the participants without wearing an activity monitor.

The lifestyle interventions during both N and G periods were the same methods with the exception of wearing the activity monitor with computerized game functions. Similarly to N, wearing the activity monitor during G also seemed to confer a motivational effect on daily physical activity. Nevertheless, the step counts and amount of PA were significantly higher in G than in N (mean differences: 1,809 ± 407 steps (23.9 ± 5.6%) and 0.7 ± 0.2 METs · hour (36.5 ± 8.9%), respectively). These findings suggest that wearing an activity monitor with computerized game functions was the main contributor to a 36.5 ± 8.9% increase in physical activity above N. In addition, throughout the six weeks of intervention, daily physical activity was continuously higher in G than in N. These results indicate that the G period had more of an effect on the amount of steps and PA than N throughout the intervention period and that the results were not due to error. Also, this crossover study design allowed for comparisons of steps and amount of PA performed by the same individuals during the N and G periods. Thus, our results were substantially unaffected by inter-individual differences in daily physical activity caused by lifestyle differences among participants. Because we did not find any significant main group effects, higher levels of daily physical activity in the G period were similar, regardless of the order of interventions. The results of the present study were also unaffected by the difference in Yuuhokei game stories. Therefore, the present results suggest that lifestyle intervention using an activity monitor with computerized game functions increases daily physical activity, such as steps and amount of PA, more effectively than a normal intervention using a standard activity monitor.

Why does using an activity monitor with computerized game functions increase daily physical activity? In addition to wearing a pedometer, logging daily steps in a diary has been proven to increase daily physical activity (step counts) [8]. The reason is thought to be that individuals who know whether or not daily step goals are achieved through logging their step numbers become more motivated. Because participants often become absorbed in playing the interactive video games and perceive them as enjoyable, interventions adopting active video games or a computerized game element can remarkably promote physical activity or reduce sedentary behaviors [15–18]. In regard to lifestyle interventions using a behavior-based reward system, open-loop feedback intervention, such as using an activity monitor and TV viewing time as a reward, increases physical activity in children [13]. Thus, the increase in physical activity might result from being conscious of wearing an activity monitor and confirming the measured values associated with rewards and/or enjoyment. In particular, previous studies described that playing difficult games induces more failure experiences, thereby reducing the propensity to continue playing [35]. Yuuhokei, which was used as the activity monitor with computerized game functions in this study, is visually simple to understand and displays step counts until a goal is achieved. When subjects reach the goal of step counts (that is accumulating 8,000 steps), the story, scene, or stage of the role-playing game in Yuuhokei progresses, and then the participants can regularly access it until their clear scene and read a success story of the game in detail at any time. Moreover, the game characters in Yuuhokei usually cheer the users according to step count milestones or physical activity levels. That is, when the participants accumulate 8,000 steps counted by Yuuhokei, game characters praise the participants considerably. Conversely, when they constantly cannot accumulate 8,000 steps, game characters encourage them to increase their physical activity and to clear the next scene of the game. The games included in Yuuhokei are based on stories and tales that are very famous in Japan, and all study participants knew almost all of the game stories or tales. Thus, participants could easily become absorbed in using Yuuhokei, thereby increasing physical activity to clear the game. In this study, although the data feedback by an exercise instructor was the same frequency between the both interventions (once a week), using an activity monitor with computerized game functions is more likely to induce an increase in a frequency of confirming the measured values by participants themselves, than to use a standard activity monitor. Collectively, the game story, scenes, and characters may help to motivate the wearer to walk or be active and make them even more conscious of daily physical activity levels and the step goals. Therefore, the synergistic effects of wearing an activity monitor and using a device with a computerized game component for rewards could contribute an increase in daily physical activity, such as steps and the degree of physical activity. Further detailed investigations (that is quantifying the frequency of confirming the measured values by participants themselves) are needed to address this notion.

Ross and Janssen have shown a dose-dependent positive association between the degree of physical activity and fat loss within a relatively short intervention of 16 weeks [19]. Thus, further increases in physical activity associated with an activity monitor that includes computerized game functions should more effectively reduce body fat. In fact, body mass, body fat, and BMI were significantly reduced in both N and G. Because lean body mass did not significantly change in either interventions, the decrease in body mass after lifestyle intervention primarily resulted from a reduction in the amount of body fat. In addition, significantly more body fat was lost in G than in N. Although the baseline of physical activity and dietary intake amount are unclear in this population, the physical activity at an intensity of ≥ 3 METs during G period expended 7,958.0 ± 710.8 kcal (amount of PA (METs · hour) × body mass (kg) × 1.05 × period (6 weeks)). A body fat reduction of 1.0 kg generally needs to expend 7,000 to 8,000 kcal [18]. Because body fat changes during G period were −0.8 ± 0.2 kg, the degree of fat reduction is approximately consistent with the amount of PA conducted during G period. Thus, our results support the notion that more body fat would be lost during the intervention using an activity monitor with computerized game functions than the normal standard activity monitor.

This study has several important limitations. Firstly, although the National Health and Nutrition Survey Japan in 2010 reported that step counts in Japanese people are 6,636 steps (men 7,174 steps; women 6,176 steps) [36], we could not assess the steps and physical activity amount baseline for our participants. Due to the participants coming from a small, homogenous, and healthy active sample as a pilot study, there would not have been major changes in body composition without dietary modification. In this study, no significant correlation was observed between the changes in body composition and the changes in step counts or amount of PA (data were not shown), suggesting that our experimental design may be insufficient to detect the degree of increase in daily physical activity from the baseline to the intervention period. Although further investigations are required, our results at least indicate that the intervention using an activity monitor with computerized game functions increases daily physical activity and reduces body fat more effectively than that using only a normal activity monitor. Secondly, this crossover study could not include a washout period between G and N. Group A (6 weeks of G followed by 6 weeks of N) might have had lingering effects of wearing the activity monitor with computerized game functions until the day before starting the 6-week N period. Thus, the findings should be interpreted with caution. Finally, our findings give no information about whether or not the effects of activity monitor with computerized game functions is globally universal, because this content of the activity monitor is based on Japanese specific culture. To elucidate it, further studies and development of devices are need.

## Conclusions

The present results indicate that the lifestyle intervention using an activity monitor with computerized game functions increases daily physical activity, such as steps and physical activity amount, more effectively, and thus causes the loss of more body fat than intervention using a normal activity monitor. These findings therefore have important implications for achieving goals such as 10,000 steps/day more easily or ensuring compliance with the *Physical Activity Reference for Health Promotion 2013* [4].

## Abbreviations

ACSM: American College of Sports Medicine
AHA: American Heart Association
ANOVA: analysis of variance
Amount of PA: amount of physical activity
BP: blood pressure
BMI: body mass index
CV: coefficients of variations
G: game intervention
JIS: Japanese Industrial Standards
METs: metabolic equivalents
N: normal intervention
PC: personal computer
SEM: standard error of the mean.
